# Disease Risk Perception and Safety Practices: A Survey of Australian Flying Fox Rehabilitators

**DOI:** 10.1371/journal.pntd.0004411

**Published:** 2016-02-01

**Authors:** Cecilia A. Sánchez, Michelle L. Baker

**Affiliations:** 1 Odum School of Ecology, University of Georgia, Athens, Georgia, United States of America; 2 CSIRO, Australian Animal Health Laboratory, Health and Biosecurity Business Unit, Geelong, Victoria, Australia; The American Humane Association, UNITED STATES

## Abstract

Interactions with flying foxes pose disease transmission risks to volunteer rehabilitators (carers) who treat injured, ill, and orphaned bats. In particular, Australian bat lyssavirus (ABLV) can be transmitted directly from flying foxes to humans in Australia. Personal protective equipment (PPE) and rabies vaccination can be used to protect against lyssavirus infection. During May and June 2014, active Australian flying fox carers participated in an online survey (SOAR: Survey Of Australian flying fox Rehabilitators) designed to gather demographic data, assess perceptions of disease risk, and explore safety practices. Responses to open-ended questions were analysed thematically. A logistic regression was performed to assess whether rehabilitators’ gender, use of PPE, threat perception, and years of experience predicted variation in their odds of being bitten or scratched. Eligible responses were received from 122 rehabilitators located predominantly on the eastern coast of Australia. Eighty-four percent of respondents were female. Years of experience ranged from <1 to 30 years (median 5 years). Respondents were highly educated. All rehabilitators were vaccinated against rabies and 94% received a rabies titre check at least every two years. Sixty-three percent of carers did not perceive viruses in flying foxes as a potential threat to their health, yet 74% of carers reported using PPE when handling flying foxes. Eighty-three percent of rehabilitators had received a flying fox bite or scratch at some point during their career. Carers provide an important community service by rescuing and rehabilitating flying foxes. While rehabilitators in this study have many excellent safety practices, including a 100% vaccination rate against rabies, there is room for improvement in PPE use. We recommend 1) the establishment of an Australia-wide set of guidelines for safety when caring for bats and 2) that the responsible government agencies in Australia support carers who rescue potentially ABLV-infected bats by offering compensation for PPE.

## Author Summary

Wildlife rehabilitators can encounter risks when handling animals, such as physical harm and exposure to infectious diseases. In Australia, rehabilitators that care for fruit bats may be exposed to Australian bat lyssavirus if bitten or scratched, which is fatal to people not vaccinated against rabies. We initiated a survey to assess rehabilitators’ perceptions of disease risk associated with fruit bats as well as rehabilitators’ safety practices. Despite an excellent rabies vaccination rate (100%), we found room for improvement in use of personal protective equipment. Supporting this, our regression analysis showed that use of protective equipment is associated with less chance of being bitten or scratched. Rehabilitators that are able to safely handle fruit bats can reduce risk to themselves, model good behaviour for onlookers, and protect animals from euthanasia. We recommend that carers develop Australia-wide guidelines for safety when rehabilitating bats and that the responsible government agencies in Australia support carers who rescue potentially lyssavirus-infected bats by offering compensation for costly protective equipment.

## Introduction

Mainland Australia is home to four species of *Pteropus* fruit bats (flying foxes) which play an important ecological role by pollinating forest ecosystems and dispersing seeds as they forage for nectar, pollen, and fruit [1]. Though generally hardy animals, flying foxes occasionally sustain injuries due to extreme weather events [2] or manmade hazards (e.g. barbed wire, netting, power lines) [3]. A number of volunteer rehabilitators (commonly known as carers—the terms are used interchangeably here) care for injured, ill, and orphaned flying foxes, often as part of wildlife care groups [4]. A carer typically rehabilitates a flying fox until it can be returned to the wild; in cases of debilitating or lasting injury, a flying fox is euthanised or occasionally kept in permanent care.

Of concern to human health, and rehabilitator health in particular, is that flying foxes are reservoir hosts of zoonotic viruses—those passed from an animal to a human [5]. The most prominent of these in Australia are Australian bat lyssavirus (ABLV) and Hendra virus (HeV), both of which cause fatal disease in humans [5]; however, only ABLV is known to be transmitted directly from bats to humans. All four mainland species of flying fox (the black, *Pteropus alecto*; the grey-headed, *P*. *poliocephalus*; the little red, *P*. *scapulatus*; and the spectacled, *P*. *conspicillatus*) are reservoirs of ABLV and HeV [6–8]. ABLV infection has additionally been recorded in a species of insectivorous bat [7].

The four fatal human cases of HeV to date resulted from close contact with horses [9] and there is no evidence of direct flying fox to human transmission [10]. In contrast, ABLV can be transmitted directly from an infected bat to a human via a bite, scratch, or saliva contamination of broken skin or mucous membranes [11–13]. The clinical consequences of ABLV infection mirror those of classical rabies [14]. Guidelines recommend that wound care and rabies post-exposure prophylaxis be administered following any Category II exposure (nibbling of uncovered skin, minor scratches or abrasions without bleeding) or Category III exposure (scratch, bite, or saliva contamination of broken skin or mucous membranes) [15,16]. Rabies and ABLV are among the few viruses capable of causing clinical and pathological signs of disease in bats [17,18]. ABLV-infected bats often display neurological signs of infection and aggression and are frequently unable to fly [8], thereby increasing the opportunity for interaction with humans and other animals. Since the identification of ABLV in 1996, there have been three documented human fatalities (all occurring in Queensland) including one wildlife carer who had cared for both flying foxes and insectivorous bats [11–13].

Members of the Australian public are cautioned not to handle bats [19–21]; instead, bat rehabilitators are commonly called upon to rescue bats trapped in fencing or netting. These rescues, along with daily interactions such as treating injuries and hand-feeding pups, pose a bite and scratch hazard to carers. Carers are at special risk of ABLV infection as sick, injured, and orphaned bats have a significantly higher rate of ABLV infection than healthy bats [8]. Use of personal protective equipment (PPE) is recommended for rehabilitators [15,16] and rabies pre-exposure vaccination is typically required by care organisations. While state guidelines for the care of flying foxes exist [22–26], there is no set of unifying regulations in place across Australia, and safety practices vary between carers and care organisations.

No studies to date have comprehensively assessed risk perception, safety practices, and potential disease exposure in the Australian flying fox rehabilitator community. Studies of human-bat interactions in Australia incorporating carers have focused primarily on potential ABLV exposures [27–29]. More recent studies have examined the Australian public’s knowledge of and attitudes towards bats, including risk perception, but these were not designed to target bat rehabilitators [30–33]. Only two studies have specifically characterized the rehabilitator community. After two outbreaks of HeV (then known as equine morbillivirus) in Queensland in 1994, 128 bat carers were tested for antibodies to HeV and additionally asked to report their contact history (including bites and scratches) with flying foxes [10]. The study reported that some carers were concerned about the risk of HeV infection from flying foxes, but did not provide exact numbers. A 1998 survey explored demographics and motivations of flying fox rehabilitators, with a minor focus on risk perception [4]. Neither addressed safety practices, thus information regarding this aspect of care is especially deficient.

This study addresses the current lack of information about the flying fox rehabilitator community in Australia by presenting updated demographic data, assessing disease risk perception among carers (specifically focusing on viruses), and exploring the safety practices carers employ and the reasons underlying their actions.

## Methods

### Participant recruitment

A total of 21 email addresses for Australian flying fox and wildlife care organisations, as well as wildlife health interest groups, were identified via 1) online searches using various combinations of the following keywords: “flying fox”, “bat”, “carer”, “rehabilitator”, “wildlife”, and “Australia” and 2) referral. All organisations (S1 Table) were contacted via a solicitation email containing a link to an online survey (SOAR: **S**urvey **O**f **A**ustralian flying fox **R**ehabilitators) hosted on SurveyMonkey from 8 May to 1 June 2014. The survey was open to Australian adults (aged 18 years and older) who had cared for flying foxes within the last twelve months. Participants were encouraged to share the survey link with rehabilitators unaffiliated with a care organisation.

### Survey design

The survey design was based in part on previous work [4] but was modified and updated to reflect the increased awareness of flying foxes as reservoir hosts for a variety of zoonotic viruses [34]. The survey was piloted with four individuals familiar with flying foxes but not involved in their care. Recommendations were incorporated before wider distribution to the target audience.

The survey (S1 File) included demographic questions on gender, age, state or territory of residence, level of education, and whether the respondent had ever completed a similar survey. Several questions addressed aspects of caring for flying foxes, such as motivations, years of experience, care organisation affiliation, and where flying foxes were housed while in care. Further questions focused on threat and risk perception. In the survey, “threat perception” was used to describe the implications of viral infections carried by bats on carer health, while “risk perception” was used in reference to questions relevant to the behaviour of carers to mitigate the risks associated with bites or scratches or potential exposure of pets. Carers were asked whether they felt that viruses found in flying foxes posed a potential threat to carer health. Carers also used ordered rating scales to rate the risk to human health posed by several hypothetical situations involving flying foxes. Response options ranged from “high risk” to “no risk” with an additional option of “don’t know.” Participants were questioned regarding their safety precautions, including rabies vaccination status, frequency of titre checks, whether these checks were self-initiated or required by a care organisation, preferred PPE, and whether they had ever been bitten or scratched by a flying fox. For certain multiple-choice questions participants were asked to further explain their choice in an open-ended response; participants were also provided room at the end of the survey to make additional comments.

### Data management and statistical analysis

Survey responses were exported from SurveyMonkey into Microsoft Excel as a.csv file. Responses to open-ended questions were manually spell-checked to ensure clarity between investigators, then analysed thematically [35]. Initial codes were generated and refined to classify responses; thematic maps were then created to sort codes into broader themes and sub-themes. Once themes were reviewed and refined, all responses were re-coded.

All statistical analyses were performed in the R statistical environment (version 3.1.1) [36]. A binary logistic regression was performed to determine whether rehabilitators’ gender, use of PPE, threat perception, and years of experience predicted variation in whether they had been bitten or scratched by a flying fox during their careers. The regression was performed using the *glm* function with a binomial error distribution and logit link function. Threat perception was assessed by carers’ responses to the question, “Do you feel that viruses found in flying foxes are a potential threat to the health of carers?”. Threat perception (yes/no), gender (male/female), and PPE (none/any) were categorical variables, while years of experience was included as a continuous covariate to account for the fact that rehabilitators with more experience would have had more occasions to be bitten or scratched. “Any” PPE was defined as all categories of PPE other than “nothing” (i.e. nitrile gloves, heavy gloves, or other PPE). Explanatory variables were checked for multicollinearity by calculation of variance inflation factors using the *vif* function in the *car* package [37]. Model performance was assessed by creating a receiver operating characteristic curve and calculating the area under the curve (AUC) with the *roc* function in the *pROC* package [38].

### Ethics

The study was approved by the CSIRO Health and Medical Research Human Research Ethics committee (protocol LR07/2014). Participants gave informed consent by reading a consent page and clicking a button to proceed with the survey.

## Results

### Characteristics of respondents

A total of one hundred thirty-six survey responses were received. Participants’ responses were excluded if they did not complete the survey or did not fit the eligibility requirements, leaving 122 remaining responses (S2 File). The number of eligible rehabilitators reached by the online solicitation is unknown, and thus the response rate could not be determined; however, the number of responses is comparable to similar studies [4,10].

Selected demographic characteristics of rehabilitators are displayed in Table 1. Eighty-four percent (103/122) of carers were female; half of all carers (50%, 61/122) were 45–64 years old. Most responses were received from rehabilitators residing in New South Wales (47%, 58/122), Queensland (34%, 42/122), and Victoria (11%, 14/122). Fifty-five percent (68/122) of carers listed university or technical college as their highest level of education. Almost all (94%, 115/122) respondents indicated that they were affiliated with a care organisation; in total, 36 care organisations were represented. Nearly all rehabilitators (95%, 116/122) reported that they had never participated in a similar survey. Years of experience ranged from <1 to 30 years (median 5 years; not displayed in Table 1).

**Table 1:** Demographic characteristics of 122 Australian flying fox rehabilitators, 2014.

Characteristic	%^a^	*n*
Gender		
Female	84.4	103
State/territory^b^		
ACT	2	2
NSW	47	58
NT	3	4
QLD	34	42
SA	2	2
VIC	11	14
Age group		
18–24 years	4	5
25–44 years	28	34
45–64 years	50	61
65 years and over	18	22
Highest level of education		
High school or earlier	22	27
University or technical college	55	68
Postgraduate study	22	27
Associated with a care group		
Yes	94.3	115
Have participated in similar survey		
No	95.1	116

^a^ Percentages may not equal 100% due to rounding

^b^ ACT, Australian Capital Territory; NSW, New South Wales; QLD, Queensland; SA, South Australia; VIC, Victoria.

### Motivations of participants for caring for flying foxes

To investigate their motivations for caring, participants were asked, “What do you enjoy most about caring for/handling flying foxes?” and asked to choose two options from a list developed by Markus & Blackshaw [4]. Responses to this question are presented in Table 2. Returning the flying fox to nature (67%, 82/122) and helping to conserve the species (55%, 67/122) were the most popular choices. Among carers that listed “Other” as a motivation (14%, 17/122), the main themes were public outreach and education, close interaction with flying foxes, and a desire to help animals.

**Table 2:** Motivations of 122 Australian flying fox rehabilitators, 2014

	Respondents indicating this motivation^a^	
Motivation	%	n
Returning the flying fox to nature when it is able to fend for itself	67	82
Helping to conserve the species	55	67
Being able to observe and learn about a wild flying fox	35	43
Helping the flying fox to survive	34	41
Other	14	17
Being able to nurse and care for a helpless animal	12	15
Having a temporary pet without the long-term commitments	0	0

^a^ Respondents were asked to choose two motivations, but 29 chose either one or more than two.

### Safety practices

Safety practices employed by carers are summarized in Table 3. All rehabilitators (100%, 122/122) reported that they were vaccinated against rabies, which is used to protect against ABLV infection. Most carers (94%, 115/122) reported having their rabies titre checked at least every two years; for 58% (70/122), titre checks were required by their care organisation, while 38% (46/122) of carers initiated the checks. Of the five (4%, 5/122) rehabilitators who reported never having their titres checked, four had ≤ 2 years of experience. Most rehabilitators reported that flying foxes in their care were housed in a human residence (40%, 49/122) or a human and pet residence (30%, 36/122).

**Table 3:** Reported safety practices of 122 Australian flying fox rehabilitators, 2014

Characteristic	%^a^	*n*
Vaccinated against rabies		
Yes	100	122
Frequency of titre checks		
Every 6 months	1	1
Every year	64	78
Every two years	30	36
Other	6	7
Reason for titre check^b^		
Self-initiated	38	46
Required by care organisation	58	70
Never had titre checked	4	5
Personal protective equipment used to handle flying foxes		
Nothing	26	32
Nitrile or similar gloves	10	12
Heavy gloves	16	19
Other	48	59
Where in-care flying foxes are housed		
Bat-only facility	24	29
Wildlife-only facility	7	8
Human residence	40	49
Human and pet residence	30	36

^a^ Percentages may not equal 100% due to rounding

^b^ One non-response; calculation of percentages adjusted accordingly

A little over a quarter of carers (26%, 32/122) reported that they typically used no protection to handle flying foxes. The ease of handling a flying fox was a high priority among these rehabilitators, with nearly all expressing the opinion that using gloves limited dexterity and reduced sensitivity. These limitations were felt to potentially increase the risk of a bite (e.g. due to not being able to feel the position of a flying fox’s head) or of harming the bat (e.g. by inadvertently applying excess force or pressure). Less commonly expressed was a belief that vaccination, experience, and training provided protection without a need for PPE.

Another quarter of carers (25%, 31/122) reported that they most commonly used nitrile or heavy gloves (nitrile or similar, 12/31; heavy gloves, 19/31). Of the 59 rehabilitators (48%, 59/122) who reported “Other” as their typical PPE, 18 (31%, 18/59) said that their choice of PPE depended on the situation and the flying fox being handled. Towels and blankets were the most popular alternative PPE listed; carers articulated that they provided a balance of personal safety and dexterity. Rehabilitators also reported using other types of gloves, arm protection (e.g. long sleeves, Neoprene arm protectors), and eye protection. There was a perception of low risk among carers who reported using “Other” or no PPE; it was not always specified whether this was a risk of being bitten or scratched or a risk of disease transmission.

Self-protection (e.g. against bites, scratches, and associated pain) was the most common motivation among rehabilitators who reported using some form of PPE. Protection of the bat was also a theme, with carers recognizing that receiving a bite or scratch would mean euthanasia of the bat (as guidelines for public health units recommend the testing, typically via a fluorescent antibody test on brain tissue, of any bat involved in a potential ABLV exposure [15]). Five rehabilitators reported using PPE specifically when being observed by members of the public to set a good example.

### Disease threat and risk perception associated with bats

Although bats likely harbour a variety of pathogens (bacteria, parasites and viruses), only zoonotic viruses such as ABLV have been associated with human disease and were therefore the focus of the current survey. A majority of carers (63%, 77/122) reported that they did not feel that viruses found in flying foxes were a potential threat to carer health. When asked to elaborate, the key theme was that vaccination against rabies and regular titre checks eliminated any threat. Secondary themes included the importance of training, hygiene, and handling techniques, and perceptions of low prevalence of ABLV infection in flying foxes and low chance of disease transmission. Several carers expressed that they felt it was possible to recognize bats infected with ABLV. Direct bat-to-human transmission of HeV was not perceived as a threat. Among rehabilitators that did feel that viruses were a potential threat (37%, 45/122), themes included the potential lethality of ABLV infection (including a previous carer death) the high contact rate between carers and bats compared to the general public, and the potential for flying foxes to harbour other viruses. As in the former group, rehabilitators emphasized proper handling and training. Carers in both groups perceived certain categories of flying foxes to pose more of a threat to their health, namely adults, wild-caught bats, and “odd” or “suspicious” bats.

**Fig 1:**
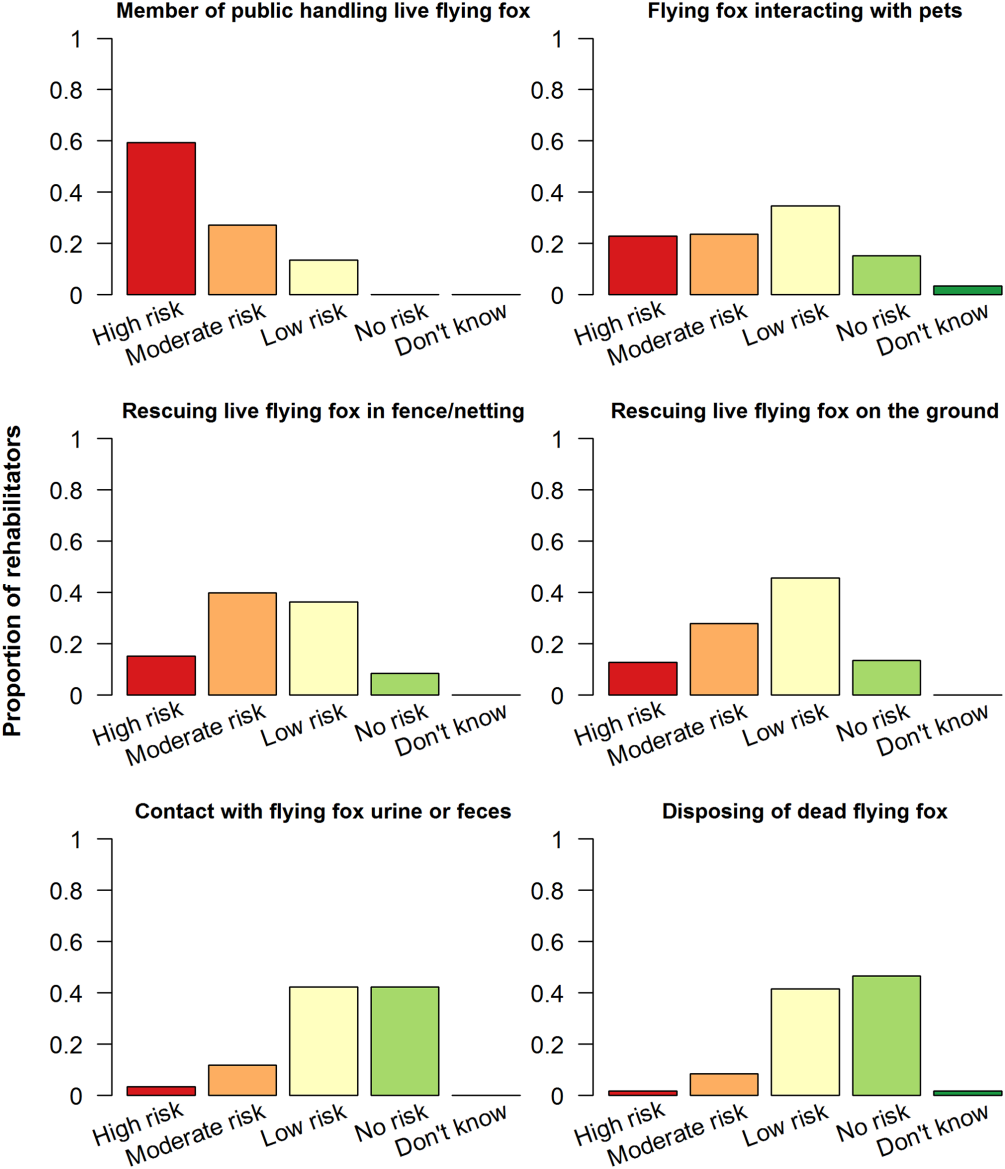
Risk to human health rated by 118 Australian flying fox rehabilitators, 2014. Rehabilitators assigned risk ratings to multiple hypothetical scenarios involving a flying fox.

Participants additionally rated the risk to human health in a number of hypothetical situations (adapted from [30]) involving flying foxes (Fig 1, S2 Table). A member of the public handling a live flying fox was perceived as the riskiest situation (59%, 70/118, rated as high risk), while disposing of a dead flying fox was perceived as the least risky situation (47%, 55/118, rated as no risk). Responses of “Don’t know” were recorded for only two situations: a flying fox interacting with pets and disposing of a dead flying fox. Four respondents failed to assign a risk to one or more scenarios and were not included in Fig 1.

### Predictors of being bitten or scratched

A binary logistic regression was performed to determine whether any groups of carers are more likely to be bitten or scratched when handling flying foxes. Specifically, this analysis examined whether carers’ use of PPE, gender, threat perception, and years of experience predicted variation in the response variable: whether a carer had been bitten or scratched by a flying fox in their career. Results of logistic regressions are typically reported in odds ratios (ORs), where an OR of 1 indicates that two groups have equal odds of experiencing an outcome of interest. An OR greater than 1 for a given group indicates that the group has higher odds of experiencing the outcome of interest than a second group. If the 95% confidence interval of the OR does not cross 1, this is generally considered a significant result [39].

**Fig 2:**
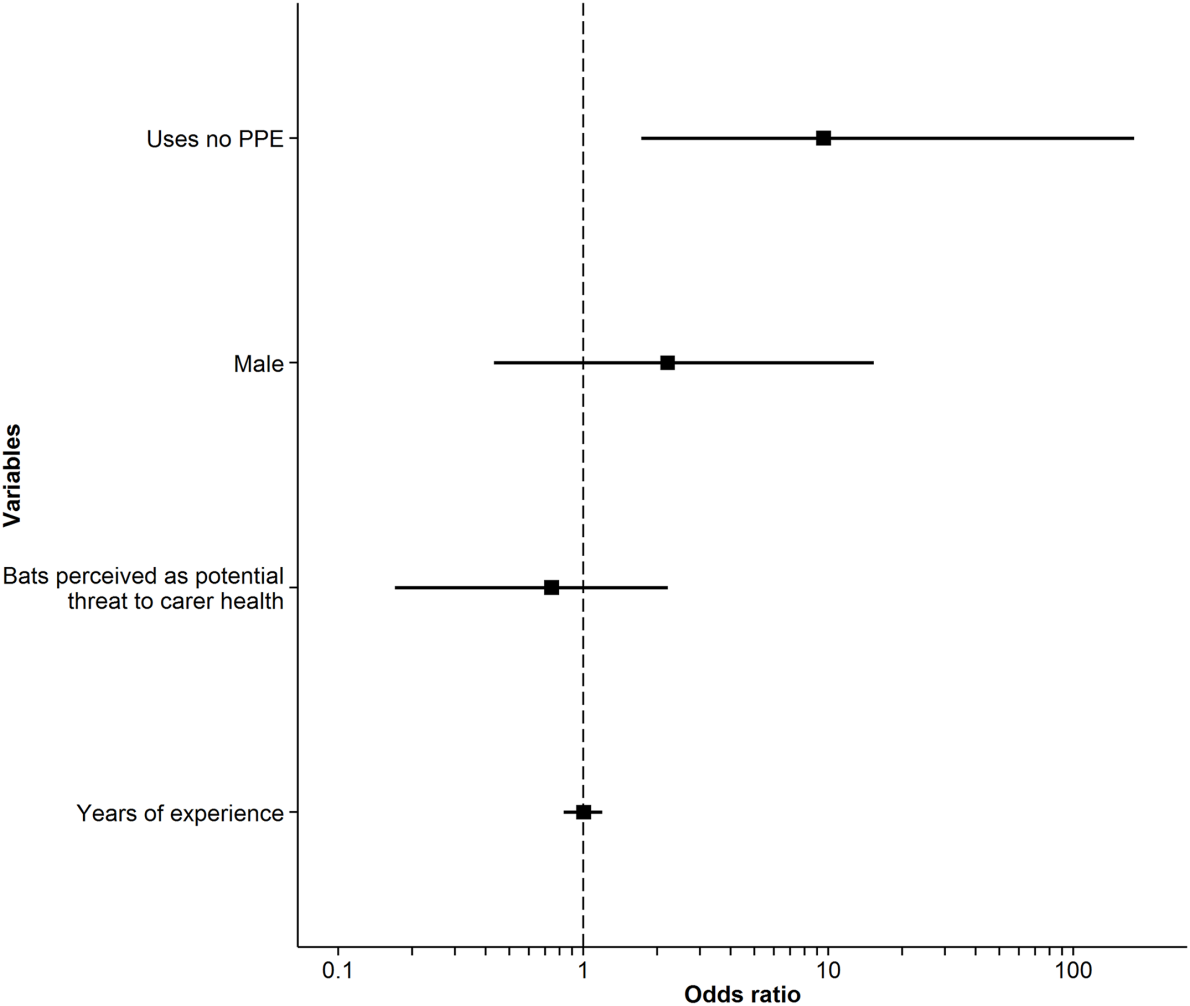
Odds ratios and 95% confidence intervals of predictors for being bitten or scratched. A 95% confidence interval that does not cross 1 is considered a significant result. The x-axis is on a log scale.

Rehabilitators who wore no PPE had 9.58 times (95% CI: 1.83–177) the odds of being bitten or scratched compared to those who used any type of PPE (nitrile gloves, heavy gloves, or other protection; Fig 2, S3 Table). Wearing heavy gloves provided the best protection, followed by nitrile gloves and other protection (S4 Table). A carer’s gender, threat perception, and years of experience did not significantly predict variation in being bitten or scratched (Fig 2, S3 Table).

### Post-exposure management

Most carers (83%, 101/122) indicated that they had been bitten or scratched by a flying fox during their career (females: 82%, 84/103; males: 89%, 17/19). The Communicable Diseases Network Australia (CDNA) recommendation for post-exposure management (PEM) comprises both wound management and receiving post-exposure prophylaxis [15]. Carers were asked to elaborate on how they responded to being bitten or scratched or how they would respond if they had not been bitten or scratched. For carers who had been bitten or scratched, this question was intended to assess actual post-exposure actions taken, but a number of carers gave hypothetical responses. Carers reported a range of PEM; at one extreme, carers reported that they would do nothing or ignore the wound, while at the other extreme, carers reported that they would wash the wound, apply an antiseptic or virucide, and receive a rabies booster shot. Other carers reported that they would practice wound care but not seek medical attention. A main theme was that carers’ responses depended on several factors, such as a bat’s health status and rescue or care history. Scratches were reported to be common, especially from orphaned flying foxes, and perceived to be less risky than bites. Some carers factored their rabies titre level or date of last vaccination into the decision to seek medical attention. Attitudes towards euthanasia of a flying fox that had bitten or scratched a rehabilitator (as per Public Health Unit guidelines) varied. While some carers reported that they would euthanise the bat themselves or contact health authorities to arrange euthanasia, others emphasised that they would never euthanise a bat, or only as a last resort.

## Discussion

One hundred twenty-two eligible responses were received for this survey, a number comparable to similar studies reported in 1998 and 2014 [4,10]. Demographic characteristics of carers in 2014 were similar to those reported in 1998 [4]; most carers were female and were between the ages of 45–64. While Markus & Blackshaw [4] did not directly ask rehabilitators’ educational level, the authors inferred from occupation that many were highly educated. Likewise, 55% (68/122) and 22% (27/122) of carers in this study listed university/technical college and postgraduate study, respectively, as their highest level of education. Rehabilitators were thus highly educated compared to the Australian population as of May 2014, of which 45.7% attained a bachelor’s degree, graduate or advanced diploma, or Certificate III/IV, and 5.2% received a postgraduate degree [40].

Compared to the 1998 survey [4], the present study had a greater geographic representation. No responses were received from Victoria, the Australian Capital Territory, Northern Territory, or South Australia in 1998, whereas rehabilitators from these states and territories made up 18% of respondents in the current survey. This may reflect increased ease of contacting carers through online methods, rather than any shift in range limits of flying foxes [41]. The high percentage of respondents from NSW and QLD is likely due to the large number of care organisations found in these two states, which in turn reflects the distribution of flying foxes along the eastern coast of Australia, and is unlikely to introduce significant bias to the results. While at least 36 care organisations are represented, the true number of organisations represented is probably higher, as some carers did not specify the organisation to which they belonged, while others indicated only an umbrella organisation rather than an individual branch.

### Disease threat and risk perception and safety practices

Since 1998, a number of novel viruses have been identified in both frugivorous and insectivorous Australian bats (e.g. Cedar virus and other paramyxoviruses, Broome virus) [42–45]. However, disease threat perception rates associated with bats amongst carers have remained moderate. A smaller percentage of carers in 2014 compared to 1998 felt viruses found in flying foxes were a potential threat to carer health (2014: 37%, 45/122; 1998: 41%, 49/119), but this difference was not statistically significant (P = 0.2862, two-tailed Fisher’s exact test). Threat perception appears to be driven by a focus on ABLV. Given the low number of fatalities due to ABLV combined with high rates of rabies vaccinations and titre checks, rehabilitators may not view potential viral transmission as a threat.

Risk ratings of hypothetical situations involving flying foxes were generally intuitive (e.g. a member of the public handling a flying fox was likely perceived as the riskiest situation because average citizens are rarely rabies-vaccinated). Two situations are of particular interest. A flying fox interacting with pets had the second-highest number of “high risk” ratings. This is important considering that 30% of rehabilitators reported that flying foxes were cared for in a human and pet environment. Disposing of a dead flying fox was perceived as the least risky situation. Although the CDNA considers contact with a flying fox that has been dead for more than four hours to be low risk [15], rabies virus has been found to remain viable for more than four hours under favourable temperature and sunlight conditions [46]. Thus, rehabilitators disposing of flying fox carcasses should still consider using PPE.

Despite moderate threat perception of viruses in flying foxes, and moderate-to-low risk perception of hypothetical rescue situations, carers reported high frequency of PPE use when handling flying foxes. This discrepancy may be due to regulations imposed by care organisations, or, as some rehabilitators indicated, a desire to model appropriate behaviour for observing members of the public. Although we asked carers to report what they “typically” used to handle flying foxes, it is possible that carers vary their PPE depending on the threat they associate with a particular bat. Adult and wild-caught bats were perceived to pose more of a threat to carers, which corresponds with data on bats submitted for ABLV testing as part of a surveillance program between June 1996 and March 2002 [8]. However, the recent detection of ABLV in three juvenile flying foxes underscores that bats of all ages can be infected [47]. While age and origin of a bat are relatively easy to identify, “odd” or “suspicious” bats were also perceived to pose an increased threat. ABLV infection can cause a range of behaviours in infected bats, from paresis (weakness) to aggression, and carers may have different thresholds in considering a bat suspicious. A carer overconfident in her or his ability to diagnose ABLV infection, or unwilling to have a bat euthanised, might postpone or forgo seeking medical attention if bitten or scratched by a flying fox.

### Potential disease exposures and post-exposure management

When carers in Queensland and New South Wales were tested for HeV antibodies in the mid-1990s, 74% reported having been bitten and 88% reported having been scratched by a flying fox [10]. These values are comparable to the 83% in this study who reported having been bitten or scratched. While this percentage does not give a sense of how many times a carer has been bitten or scratched, just a single exposure can be sufficient for ABLV infection. Our results suggest that rehabilitators should use PPE when handling flying foxes in order to reduce their odds of being bitten or scratched. However, we recognize that PPE is just one component involved in handling a bat, and that as carers emphasized, learning safe handling techniques is also important.

Carers ranged in the levels of PEM they reported, from taking no action to fully adhering to the CDNA guidelines (practicing wound care and receiving post-exposure prophylaxis). Many carers appeared to adjust their PEM based on the wound’s severity (e.g. bite or scratch), the perceived risk posed by the bat (e.g. age, behaviour), and knowledge of their rabies titre level.

### Limitations

Our results may be subject to selection bias because the survey was hosted online and was thus only available to people with internet access. There may be less representation from carers in remote areas and carers with lower socioeconomic status. In addition, because participants were recruited via email discussion lists, rehabilitators unaffiliated with a care organisation may be underrepresented. The lack of a centralized registry of carers prevents an estimate of whether the sample is representative of the carer community as a whole. Our results may also be subject to volunteer bias, as rehabilitators who have a high level of compliance with recommended safety measures may have been more likely to complete the survey. Because all experiences were self-reported, they may be subject to recall bias. Although the survey was anonymous to encourage honesty, participants may have underreported risky behaviours.

Some carers indicated that the wording of the hypothetical risk scenarios were unclear, as the risk to human health could be interpreted from the point of a carer or a member of the public. For this reason, we did not include a numeric measure of risk perception, as calculated by Young et al. [30], as an explanatory variable in the regression analyses.

### Summary and recommendations

This report describes the results of the first survey designed to explicitly gather data on disease risk perception and safety practices among Australian flying fox rehabilitators. We found that carers are highly aware of ABLV, but do not perceive viruses in general to pose a threat to their health. Rehabilitators in this study have many excellent safety practices, including a 100% vaccination rate against rabies, but there is still room for improvement in the use of PPE, both in overall use and in use of heavier-duty equipment that can offer better protection. One barrier to PPE use may be cost; since carers are typically volunteers, carers may not prioritize PPE given limited funds. PEM also presents an opportunity for improvement, as some carers report not practicing wound care or seeking post-exposure prophylaxis after a bite or scratch from a bat. We recommend the development of Australia-wide guidelines for safety when caring for bats. These guidelines, developed by carers, should emphasize the importance of proper PPE use to reduce the risk of being bitten or scratched, which will in turn protect bats from the threat of euthanasia. The guidelines should additionally provide recommendations on rabies vaccination and frequency of titre checks.

Although they work in a volunteer capacity, rehabilitators provide an important service to their communities by rescuing and rehabilitating flying foxes. Carers act as first responders in diverse situations, ranging from rescuing bats from barbed-wire fence to treating large numbers of bats in extreme heat waves, and are thus put at increased risk of zoonotic disease transmission. Bites and scratches are common in rehabilitators’ lifetimes, although this study did not measure how frequently these occurred. Given that Australian state and territory government agencies recommend that members of the public should rely on carers to handle bats [19–21], we recommend that these agencies in turn support carers. Through the HeV PPE rebate program, Queensland veterinarians are offered compensation for the cost of initial purchases of PPE, as well as for PPE used during an HeV investigation [48]. Similarly, state and territory government agencies could offer compensation to carers who rescue a potentially ABLV-infected bat. Such a program would help protect rehabilitators, and thus enable them to continue caring for the bats that are so vital to Australia’s forest ecosystem.

## Supporting Information

S1 File SOAR: Survey Of Australian flying fox Rehabilitators. (PDF)

S2 File Survey data. Responses to the survey from 122 Australian flying fox rehabilitators. Data were de-identified by removing the following information: gender, age, education level, state of residence, years of experience, and wildlife care organisation.
(CSV)

S1 Table Wildlife and flying fox organisations contacted. (DOCX)

S2 Table Risk to human health rated by 118 Australian flying fox rehabilitators, 2014: %^a^ and (n). Rehabilitators assigned risk ratings to multiple hypothetical scenarios involving a flying fox.
(DOCX)

S3 Table Results of a binary logistic regression to identify predictors of being bitten or scratched. Values are reported for β (beta) coefficient, SE (standard error), OR (odds ratio) and 95% CI (confidence interval). PPE, personal protective equipment; Threat, whether a carer considers viruses in flying foxes to be a threat to carer health. “Any form” of PPE includes nitrile gloves, heavy gloves, or other PPE. Model AUC = 0.69.
(DOCX)

S4 Table Results of a binary logistic regression to identify predictors of not being bitten or scratched. Values are reported for β (beta) coefficient, SE (standard error), OR (odds ratio) and 95% CI (confidence interval). PPE, personal protective equipment; Threat, whether a carer considers viruses in flying foxes to be a threat to carer health. Model AUC = 0.77.
(DOCX)
