# Supplementary material for: Disease Risk Perception and Safety Practices: A Survey of Australian Flying Fox Rehabilitators
Source: PLoS Negl Trop Dis. 2016 Feb 1;10(2):e0004411. doi: 10.1371/journal.pntd.0004411 (PMC4734781; doi:10.1371/journal.pntd.0004411)
Supplement: S1 File — (PDF) [file pntd.0004411.s001.pdf]

1. Consent Page

CSIRO Animal, Food and Health Sciences
LOW RISK REVIEW PANEL NUMBER: LR07/2014

INTRODUCTION
Flying foxes play an important ecological role in Australia by pollinating forest ecosystems and dispersing seeds. These generally hardy animals occasionally sustain injuries (often due to manmade hazards) or illness. A dedicated network of flying fox carers across Australia help to rehabilitate orphaned, injured or sick animals and return them to the wild. As a community, flying fox carers play a vital role but are poorly understood. We are interested in gathering basic demographic information about current flying fox carers. We are also interested in examining the safety habits of carers given that flying foxes can harbor viruses capable of being transmitted to humans. We do not wish to cause undue fear of flying foxes, which are often maligned and misunderstood. Rather, our goal is to work together with flying fox carers to best ensure their safety and enable them to continue aiding these remarkable and important animals.

This study is being conducted by Ms. Cecilia Sanchez and Dr. Michelle Baker as part of ongoing research on flying foxes at the Australian Animal Health Laboratory.

WHAT IS THE AIM OF THIS STUDY?
The aim of this study is to investigate flying fox carers' risk perceptions and safety precautions in various situations involving flying foxes.

CAN I PARTICIPATE?
We are looking for Australian residents aged 18 years and older **who have cared for one or more flying foxes within the past 12 months**. In order to complete the online survey, you will need access to a computer with internet facilities and to be able to read and understand English.

HOW WILL THE STUDY BE CARRIED OUT?
Your participation in this study will involve:
• A one-time online survey accessed through SurveyMonkey
• This survey will be self-administered – you can do it in your own time without help
• To start, you will need to click on the next button and this will take you to the survey
• Once you start, the survey should take between 15 – 20 minutes to complete
• The survey must be completed in one sitting

WHAT ARE THE BENEFITS OF PARTICIPATING IN THE STUDY?
You may not benefit directly from participation in this study, but you will be providing a valuable contribution to our understanding of carers and their perceptions to disease transmission from flying foxes. This information will guide us in providing information to current and future carers that will assist in assuring their health and safety. This information may also assist in improving public awareness of flying foxes and the importance of flying fox carers in the rehabilitation of sick and injured animals and caring for orphaned animals. Your personal results will not be available to you as the data collected are non-identifiable; however, the overall research results will be made available to all participants upon completion of the study in the form of a published paper.

ARE THERE ANY RISKS INVOLVED?
As a low-risk study, there are no foreseen physical or psychological risks involved. There may be a small inconvenience from the time taken to complete the survey; however, you are contributing to valuable scientific research. You may experience some fear of disclosing your personal information, however the data collected is anonymous and will be used at a group level only.

All human research undertaken by the CSIRO must comply with the values, principles, governance and review process specified in the NH&MRC National Statement on Ethical Conduct in Human Research (2007). A copy of the National Statement can be found at www.nhmrc.gov.au/guidelines/ethics/human_research/index.htm

HOW WILL MY PRIVACY BE PROTECTED?
CSIRO is governed under the Privacy Act 1988 (Cth). CSIRO is collecting your personal information for the purposes of conducting the study and related scientific research. CSIRO will only use and disclose your personal information in accordance with the Privacy Act 1988 and the NH&MRC National Statement on Ethical Conduct in Human Research (2007) as amended from time to time, and as otherwise required by law.
The data collected during this study are non-identifiable (anonymous).
In relation to studies conducted by CSIRO, it is customary for all personal information to be identified by a code and stored on secure CSIRO servers for a period of 7 years. Except where otherwise required by law or a government body, at the end of this period your records will be destroyed or permanently de-identified.
We will not use or disclose your information for direct marketing purposes.
CSIRO may publish study results and data in research publications and press releases; however, CSIRO will de-identify any personal information contained in the data and results so that you cannot be identified.
Where third parties are assisting CSIRO in relation to the conduct of this study (such as university staff, students and other health professionals), we may disclose your personal information to those third parties for this purpose on a confidential basis. CSIRO will require such third parties to keep this information confidential and to only use your personal information for the purposes of the study and otherwise in accordance with the Privacy Act 1988.

WHAT IF I WISH TO WITHDRAW?
You are free to withdraw at any time during the study. If you choose to withdraw (i.e. exit the survey without clicking the “Done” button), none of the information you have provided will be retained.

IF YOU HAVE FURTHER QUESTIONS
Please call Michelle Baker on 03 5227 5052 or via email: michelle.baker@csiro.au

This study has been approved by the CSIRO Animal, Food and Health Sciences Human Research: Low Risk Review Panel (CAFHS LRRP). If you would like to speak with someone with respect to ethical matters, the Human Research Ethics Co-ordinator can be contacted via email at cafhshrec@csiro.au.

If you wish to register a formal complaint about the conduct of this research project please contact the Office of the Centre Manager, CSIRO Animal, Food and Health Sciences, PO Box 10041, Adelaide BC, SA 5000 or via email (peter.royle@csiro.au).

CONSENT
If you are happy with the information provided, please indicate this below and click ‘Next’ to continue to the survey. By doing this, you are indicating your consent to participate in the study.

If you do not wish to participate in the survey, it will help us for response rate calculations if you could indicate this below and click ‘Next.’ The survey will then be terminated.

1. Do you wish to participate?

- ◯ I am eligible for this survey and wish to participate.
- ◯ I am eligible for this survey and choose not to complete it.

2. Demographic Information

2. Gender

- ◯ Female
- ◯ Male

3. Age

- ◯ 18-24 years
- ◯ 25-44 years
- ◯ 45-64 years
- ◯ 65 years and older

4. Highest level of education

- ◯ High school level or earlier
- ◯ University/technical college level
- ◯ Postgraduate studies (MA, PhD, MD, DVM etc)

5. State/territory of residence

[ ]

3. Care of flying foxes

6. What do you enjoy most about caring for/hand-rearing a flying fox? (Choose two)

- [ ] Being able to observe and learn about a wild flying fox
- [ ] Being able to nurse and care for a helpless animal
- [ ] Helping the flying fox to survive
- [ ] Helping to conserve the species
- [ ] Returning the flying fox to nature when it is able to fend for itself
- [ ] Having a temporary pet without the long-term commitments
- [ ] Other (please specify)

7. Approximately how many years of experience do you have in caring for flying foxes?

8. Are you affiliated with a wildlife care organization?

- ◯ Yes
- ◯ No

If yes, which one?

9. Where are in-care flying foxes primarily housed?

- ◯ Bat-only facility
- ◯ Wildlife-only facility
- ◯ Human residence
- ◯ Human and pet residence

4. Safety

10. Do you feel that viruses found in flying foxes are a potential threat to the health of carers?

- ◯ Yes
- ◯ No

Please explain:

11. Have you been vaccinated against rabies?

- ◯ Yes
- ◯ No

12. If you are vaccinated against rabies, approximately how often do you get your titer checked?

- ◯ Every six months
- ◯ Every year
- ◯ Every two years
- ◯ Other (please specify)

13. Choose the statement that best applies to you:

- ◯ I initiate getting my titer checked
- ◯ Titer checks are required by my care organization
- ◯ I have never gotten my titer checked

14. Evaluate the risk to human health posed by the following scenarios.

	High risk	Moderate risk	Low risk	No risk	Don't know
Rescuing a live flying fox trapped in a fence or netting	○	○	○	○	○
Rescuing a live flying fox on the ground	○	○	○	○	○
Member of public handling a live flying fox	○	○	○	○	○
Flying fox interacting with pets	○	○	○	○	○
Disposing of a dead flying fox	○	○	○	○	○
Contact with flying fox urine or feces	○	○	○	○	○

15. When handling a flying fox, what protection do you typically wear?

- ○ Nothing
- ○ Nitrile (or similar) gloves
- ○ Heavy gloves
- ○ Other (please specify)

16. Why do you choose this level of protection?

17. Have you ever been bitten or scratched by a flying fox?

- ○ Yes
- ○ No

18. If you were to be/have been bitten or scratched by a flying fox, how would/did you respond?

5. Final questions

19. Have you ever participated in a similar survey?

- ◯ No
- ◯ Yes (Can you provide any details?)

20. Is there any additional information you would like to provide?
