# Supplementary material for: Disease Risk Perception and Safety Practices: A Survey of Australian Flying Fox Rehabilitators
Source: PLoS Negl Trop Dis. 2016 Feb 1;10(2):e0004411. doi: 10.1371/journal.pntd.0004411 (PMC4734781; doi:10.1371/journal.pntd.0004411)
Supplement: S1 Table — (DOCX) [file pntd.0004411.s003.docx]

| **Organisation** |
| --- |
| Adelaide Bat Care |
| Australasian Bat Society |
| Australian Bat Clinic |
| Bat Conservation & Rescue QLD Inc. |
| Bats Qld (Flying Foxes & Microbats) Inc. |
| FAUNA (Fostercare of Australia’s Unique Native Animals) |
| Fauna Rescue of South Australia Inc. |
| Fourth Crossing Wildlife |
| North Queensland Wildlife Care Inc. |
| Ozark – Australian Wildlife Carers Network |
| RSPCA Queensland |
| Shoalhaven Bat Clinic |
| Stickee Batz |
| Sydney Wildlife |
| Tolga Bat Hospital |
| Wildcare Australia |
| Wildcare NT |
| Wildlife Assistance & Information Foundation |
| Wildlife Health Australia (formerly Australian Wildlife Health Network) |
| Wildlife Victoria |
| WIRES (Wildlife Information, Rescue and Education Service) |
