# Supplementary material for: Disease Risk Perception and Safety Practices: A Survey of Australian Flying Fox Rehabilitators
Source: PLoS Negl Trop Dis. 2016 Feb 1;10(2):e0004411. doi: 10.1371/journal.pntd.0004411 (PMC4734781; doi:10.1371/journal.pntd.0004411)
Supplement: S2 Table — Rehabilitators assigned risk ratings to multiple hypothetical scenarios involving a flying fox. (DOCX) [file pntd.0004411.s004.docx]

| **Scenario** | **High risk** | **Moderate risk** | **Low risk** | **No risk** | **Don’t know** |
| --- | --- | --- | --- | --- | --- |
| Member of public handling a live flying fox | 59 (70) | 27 (32) | 14 (16) | 0 (0) | 0 (0) |
| Flying fox interacting with pets | 23 (27) | 24 (28) | 35 (41) | 15 (18) | 3 (4) |
| Rescuing a live flying fox trapped in a fence or netting | 15 (18) | 40 (47) | 36 (43) | 8.5 (10) | 0 (0) |
| Rescuing a live flying fox on the ground | 13 (15) | 28 (33) | 46 (54) | 14 (16) | 0 (0) |
| Contact with flying fox urine or feces | 3 (4) | 12 (14) | 42 (50) | 42 (50) | 0 (0) |
| Disposing of a dead flying fox | 2 (2) | 8.5 (10) | 42 (49) | 47 (55) | 2 (2) |

^a^ Percentages may not equal 100% due to rounding
