# Supplementary material for: Disease Risk Perception and Safety Practices: A Survey of Australian Flying Fox Rehabilitators
Source: PLoS Negl Trop Dis. 2016 Feb 1;10(2):e0004411. doi: 10.1371/journal.pntd.0004411 (PMC4734781; doi:10.1371/journal.pntd.0004411)
Supplement: S3 Table — Values are reported for β (beta) coefficient, SE (standard error), OR (odds ratio) and 95% CI (confidence interval). PPE, personal protective equipment; Threat, whether a carer considers viruses in flying foxes to be a threat to carer health. “Any form” of PPE includes nitrile gloves, heavy gloves, or other PPE. Model AUC = 0.69. (DOCX) [file pntd.0004411.s005.docx]

| **Variable** |  | **β** | **SE** | **OR** | **95% CI** |
| --- | --- | --- | --- | --- | --- |
| PPE |  |  |  |  |  |
|  | Any form | Reference | -- | -- | -- |
|  | Nothing | 2.26 | 1.05 | 9.58 | 1.83 - 177 |
| Sex |  |  |  |  |  |
|  | Female | Reference | -- | -- | -- |
|  | Male | 0.79 | 0.82 | 2.21 | 0.53 - 15.2 |
| Threat |  |  |  |  |  |
|  | No | Reference | -- | -- | -- |
|  | Yes | -0.30 | 0.52 | 0.74 | 0.27 - 2.11 |
| Years of experience |  | <0.01 | 0.04 | 1.01 | 0.93 - 1.10 |
