# Supplementary material for: Disease Risk Perception and Safety Practices: A Survey of Australian Flying Fox Rehabilitators
Source: PLoS Negl Trop Dis. 2016 Feb 1;10(2):e0004411. doi: 10.1371/journal.pntd.0004411 (PMC4734781; doi:10.1371/journal.pntd.0004411)
Supplement: S4 Table — Values are reported for β (beta) coefficient, SE (standard error), OR (odds ratio) and 95% CI (confidence interval). PPE, personal protective equipment; Threat, whether a carer considers viruses in flying foxes to be a threat to carer health. Model AUC = 0.77. (DOCX) [file pntd.0004411.s006.docx]

| **Variable** |  | **β** | **SE** | **OR** | **95% CI** |
| --- | --- | --- | --- | --- | --- |
| PPE |  |  |  |  |  |
|  | None | Reference | -- | -- | -- |
|  | Nitrile gloves | 2.70 | 1.25 | 14.8 | 1.55 - 337 |
|  | Heavy gloves | 3.45 | 1.16 | 31.6 | 4.54 - 657 |
|  | Other | 1.80 | 1.08 | 6.06 | 1.05 - 115 |
| Sex |  |  |  |  |  |
|  | Male | Reference | -- | -- | -- |
|  | Female | 1.08 | 0.83 | 2.94 | 0.68 - 20.8 |
| Threat |  |  |  |  |  |
|  | No | Reference | -- | -- | -- |
|  | Yes | 0.49 | 0.55 | 1.63 | 0.54 - 4.88 |
| Years of experience |  | 0.03 | 0.04 | 1.03 | 0.94 - 1.11 |
